# Association of Household Deprivation, Comorbidities, and COVID-19 Hospitalization in Children in Germany, January 2020 to July 2021

**DOI:** 10.1001/jamanetworkopen.2022.34319

**Published:** 2022-10-03

**Authors:** Nico Dragano, Olga Dortmann, Jörg Timm, Matthias Mohrmann, Rosemarie Wehner, Christoph J. Rupprecht, Maria Scheider, Ertan Mayatepek, Morten Wahrendorf

**Affiliations:** 1 Institute of Medical Sociology, Centre for Health and Society, University Hospital and Medical Faculty, University of Duesseldorf, Germany; 2 Department of Health Management, Allgemeine Ortskrankenkasse Rhineland/Hamburg–Die Gesundheitskasse, Duesseldorf, Germany; 3 Institute of Virology, Heinrich Heine University, University Hospital and Medical Faculty, University of Duesseldorf, Germany; 4 Allgemeine Ortskrankenkasse Rhineland/Hamburg–Die Gesundheitskasse, Duesseldorf, Germany; 5 Department of Health Policy and Health Economics, Allgemeine Ortskrankenkasse Rhineland/Hamburg – Die Gesundheitskasse, Duesseldorf, Germany; 6 Department of General Pediatrics, Neonatology and Pediatric Cardiology, University Hospital Duesseldorf, Medical Faculty, Heinrich-Heine-University Duesseldorf, Duesseldorf, Germany

## Abstract

**Importance:**

Adults in disadvantaged socioeconomic positions have elevated risks of a severe course of COVID-19, but it is unclear whether this holds true for children.

**Objective:**

To investigate whether young people from disadvantaged households have a higher risk of COVID-19 hospitalization and whether differences were associated with comorbidities that predispose children to severe courses.

**Design, Setting, and Participants:**

This population-based cohort study included all children and adolescents (aged 0-18 years) who were enrolled in a statutory health insurance carrier in Germany during the observation period of January 1, 2020, to July 13, 2021. Logistic regressions were calculated to compare children from households with and without an indication of poverty. Age, sex, days under observation, nationality, and comorbidities (eg, obesity, diabetes) were controlled for to account for explanatory factors.

**Exposures:**

Disadvantage on the household level was assessed by the employment status of the insurance holder (ie, employed, long- or short-term unemployed, low-wage employment, economically inactive). Socioeconomic characteristics of the area of residence were also assessed.

**Main Outcomes and Measures:**

Daily hospital diagnoses of COVID-19 (*International Statistical Classification of Diseases and Related Health Problems, Tenth Revision *codes U07.1 and U07.2) were recorded. Comorbidities were assessed using inpatient and outpatient diagnoses contained in the insurance records.

**Results:**

A total of 688 075 children and adolescents were included, with a mean (SD) age of 8.3 (5.8) years and 333 489 (48.4%) female participants. COVID-19 hospital diagnosis was a rare event (1637 participants [0.2%]). Children whose parents were long-term unemployed were 1.36 (95% CI, 1.22-1.51) times more likely than those with employed parents to be hospitalized. Elevated odds were also found for children whose parents had low-wage employment (odds ratio, 1.29; 95% CI, 1.05-1.58). Those living in low-income areas had 3.02 (95% CI, 1.73-5.28) times higher odds of hospitalization than those in less deprived areas. Comorbidities were associated with hospitalization, but their adjustment did not change main estimates for deprivation.

**Conclusions and Relevance:**

In this cohort study, children who had parents who were unemployed and those who lived in low-income areas were at higher risk of COVID-19 hospitalization. This finding suggests that attention must be paid to children with SARS-CoV-2 from vulnerable families and closer monitoring should be considered. A number of explanatory factors, including comorbidities, were taken into account, but their analysis yielded no clear picture about underlying processes.

## Key Points

**Question:**

Do young people from households with low incomes have a higher risk of COVID-19 hospitalization compared with their more affluent counterparts, and if so, what factors are associated with this difference?

**Findings:**

In this cohort study of 688 705 children and adolescents, elevated odds of a COVID-19 hospitalization were observed among children and adolescents from households with unemployed parents and who were living in areas with lower median incomes. A number of explanatory factors, including comorbidities, were taken into account, but their analysis yielded no clear picture about underlying processes.

**Meaning:**

These findings suggest that greater attention must be paid to a possible severe course of disease in children from families with lower socioeconomic status and closer monitoring should be considered.

## Teaser

This cohort study investigates whether young people from disadvantaged households have a higher risk of COVID-19 hospitalization compared with more affluent counterparts.

## Introduction

The incidence of COVID-19 among children and adolescents was high throughout the pandemic, with a particular peak during the Omicron wave.^1,2,3^ Although the risk of falling seriously ill has been shown to be significantly lower in younger compared with older persons, some children have had critical COVID-19 courses.^4,5^ The factors associated with disease severity among children and adolescents, however, are still under research. Apart from biomedical factors, family-level socioeconomic characteristics may be important because social inequalities have been associated with all stages of the COVID-19 clinical course from infection risk^6,7,8,9,10,11,12,13,14^ to COVID-19 hospitalization^8,14,15,16^ and mortality.^8,17,18,19^

Most studies, however, are based on either adults only or aggregate data without age differentiation. Since it is unclear whether results from studies with older populations are generalizable to young people, data specific to this age group must be used.^20,21^ However, only a small number of studies have investigated inequalities in infections among the young, mostly using aggregate measures of socioeconomic position (SEP).^22,23,24^ Evidence regarding severe courses of COVID-19 is also limited to a few studies with proxy measures of SEP. A case-control study from the United States^25^ found an overrepresentation of children from low SEP neighborhoods among severe cases (measured as a presence of multisystem inflammatory syndrome), while another US-based study^26^ found no association between SEP and COVID-19 severity among pediatric patients already hospitalized. Furthermore, 2 cohort studies from England^24,27^ reported slightly higher hospital and intensive care unit admission rates among children from deprived areas, and an ecological analysis from Brazil^28^ revealed higher COVID-19 mortality rates in deprived regions.

The well-documented inequalities in preexisting health conditions, such as diabetes or cardiovascular disease, have been named as core explanatory pathways in adult populations.^16,18,29,30^ The risk of a severe clinical course of COVID-19 has been shown to also increase in children if chronic conditions, such as obesity or type 1 diabetes, are present.^31,32^ At the same time, there is evidence that at least some predisposing diseases (eg, obesity or asthma) may occur more often in children from socioeconomically deprived families.^33,34^ Whether this etiological pathway is plausible has not yet been investigated.

In summary, the evidence of socioeconomic differences in severe COVID-19 clinical courses among children is limited, and the potential mechanisms remain unknown. Accordingly, this study analyzed the associations between deprivation and risk of a COVID-19–related hospitalization among children and adolescents in Germany. In addition, we evaluated whether such an association was independent of comorbidities.

## Methods

### Study Design and Population

This is a register-based cohort study using administrative data from the German statutory health insurance. We used data from one large carrier, the Allgemeine Ortskrankenkasse (AOK) Rhineland/Hamburg–Die Gesundheitskasse, which mainly insures individuals living in the regions of Rhineland and Hamburg, Germany. Of the 3 079 430 total insured members at the time of closure of the database (July 13, 2021), 690 115 individuals were aged 0 to 18 years and were insured for at least 1 day in the observation period (January 1, 2020, to July 13, 2021). A total of 1410 participants living outside Germany were excluded due to missing area-level information. Thus, the net sample comprised 688 705 observations.

Insurance carriers in Germany record sociodemographic characteristics, hospitalizations, medication, and other medical data as part of their routine data collection. These data can be used for scientific purposes even without individual informed consent. The data set was pseudonymized before analysis, and all calculations were conducted by the AOK as the official data owner. The analysis plan was approved by the data protection office of the AOK Rhineland/Hamburg and the ethics committee of the Medical Faculty of the University of Duesseldorf. This study was conducted in compliance with the Declaration of Helsinki and its amendments,^35^ and the reporting followed the Strengthening the Reporting of Observational Studies in Epidemiology (STROBE) reporting guideline.

### Measures

#### COVID-19 Hospitalization

Hospitals transfer patients’ main and secondary diagnoses to the insurance carrier as part of a regular daily data exchange system. The coding of diagnoses is usually done by the physician in charge using World Health Organization (WHO) *International Statistical Classification of Diseases and Related Health Problems, Tenth Revision, Germany *(*ICD-10-GM*) categories. In accordance with previous studies, cases were defined by either WHO *ICD-10-GM* code U07.1 (laboratory-confirmed COVID-19) or U07.2 (symptom-based, negative, or no laboratory test COVID-19).^19^ If a patient had more than 1 hospitalization with a COVID-19 diagnosis, only the first episode was considered.

#### Individual-Level Household SEP

The main exposure was household SEP classified by the legal employment status of either the parent whose health insurance covered the child or the adolescent themself if they were enrolled as a full member of the insurance carrier. Most children and adolescents in Germany are insured as part of their parents’ insurance until they finish secondary education. After reaching the age of 14 years, young people who start gainful employment or vocational training or have their income paid by social security become full members of the insurance carrier. Most children in our sample were insured by a parent (637 741 [92.6%]), and 50 964 (7.4%) were individual members of the insurance carrier.

The employment status of the insurance holder is documented using a standard coding system. Since the status information is time-varying, it was recorded at the point of study entry (ie, beginning of the study period, beginning of the insurance, birth). We distinguished 5 household SEPs: insurance holder (1) has regular employment, (2) has low-wage employment (wage less than poverty line) with additional social benefits, (3) receives short-term unemployment benefits, (4) receives social benefits for long-term unemployment, and (5) is economically inactive, ie, students, retired early, or otherwise economically inactive.

#### Area-Level Socioeconomic Characteristics

Previous studies have shown that neighborhood characteristics are associated with COVID-19; therefore, we additionally included area-level information. Indicators were linked using the postal code of the participant’s home address. Linkage was performed on the level of the 400 German districts ((Nomenclature des Unités Territoriales Statistiques–3 level^36^), and 3 indicators derived from official statistics were assigned: (1) degree of urbanization of the community of residence, (2) mean living space (in meters squared) per inhabitant in the district of residence, and (3) median income (in euros) in the district of residence. Areas were classified according to their relative position in the distribution of the respective indicator in Germany using quartiles.

#### Comorbidities

A set of diagnoses supposed to be associated with COVID-19 severity were identified in the data set using *ICD-10-GM* 2019 diagnostic codes for inpatient and outpatient health care delivery. The following diagnoses were included: obesity and other hyperalimentation (*ICD-10-GM* 2019 codes E65-E68), diabetes types 1 and 2 (*ICD-10-GM* 2019 codes E10-E14), congenital malformations of the circulatory system (*ICD-10-GM* 2019 codes Q20-Q28), neoplasms (*ICD-10-GM* 2019 codes C00-D48), asthma (*ICD-10-GM *2019 code J45), and use of immunosuppressants. The latter was measured by prescriptions for medication belonging to Anatomical Therapeutic Chemical class L04A.

A preexisting disease was considered present if the individual had at least 1 diagnosis in the years 2019 to 2021. All diagnoses from inpatient and outpatient care were available for 2019 and 2020; however, due to legal restrictions, data from 2021 relied on inpatient care only. In addition to the single diagnosis, a sum score for the number of comorbidities was computed (0, 1, ≥2).

#### Additional Variables

We included age and sex as possible confounders or modifiers in our analyses. Age (in years) was measured at the first day of insurance during the observation period for participants without an event and at the day of diagnoses for case participants. We divided the cohort into 6 age groups for descriptive and stratified analyses: 0 to 28 days, 29 days to 12 months, 1 to 5 years, 6 to 11 years, 12 to 15 years, and 16 to 18 years. The time at risk was included as an additional control variable. It was defined as the number of days from entry to the cohort until exit. Exit date was the date of diagnosis for case participants; control participants were censored at the last day of the period or the last day insured. Nationality was measured by distinguishing German vs non-German citizenship.

### Statistical Analysis

COVID-19 cases were counted, and total and group-specific cumulative incidence rates were calculated. Logistic regression models were then applied. The binary outcome was a variable indicating a hospitalization for COVID-19 in the observation period (no and yes). The outcome had a low incidence in the study cohort and fulfilled the rare disease assumption. Therefore, odds ratios (ORs) should approximate relative risks closely. Regressions were conducted with different adjustment sets. Starting with a crude model for the main exposure (individual-level SEP), adjustments were made for age (continuous and quadratic), sex, and time at risk. We next added area-level income, mean living space, degree of urbanization, and nationality of the child. A final model additionally included comorbidities.

Two sensitivity analyses were carried out. First, in addition to the joint analyses of *ICD-10-GM* codes, an analysis using laboratory confirmed cases (code U07.1) only was performed to control for a possible misclassification bias related to U07.2 codes. Second, we conducted stratified analyses for sex, age groups, and number of comorbidities to check whether interactions were present. Formal interaction was then tested using the full sample and a multiplicative interaction term for each of the 3 variables. All calculations were done with SAS Enterprise Guide 7.15.

## Results

A total of 688 075 children and adolescents (333 489 [48.4%] female; 355 216 [51.6%] male), with a mean (SD) age of 8.3 (5.8) years were analyzed. A COVID-19 hospital diagnosis was a rare event in this cohort. We recorded 1637 cases (of which 655 had U07.1 codes, and 998 had U07.2 codes) from January 1, 2020, to July 13, 2021, which corresponds to a cumulative incidence of 237.7 cases per 100 000. Our study participants had mean (SD) of 521.8 (107.3) days under observation during this time. A detailed description of the study characteristics is included in Table 1.

**Table 1.** Sample Characteristics, Including Numbers and Rates of COVID-19 Hospitalizations, From January 1, 2020, to July 3, 2021 Among 688 705 Children and Adolescents Enrolled With a Mandatory Health Insurance Carrier in Germany

Characteristic	Observations, No. (%)	Cumulative cases with COVID-19 hospitalization, No.^a^	COVID-19 hospitalization, rate per 100 000 population^b^
Total	688 705 (100.0)	1637	237.7
Sex			
Female	333 489 (48.4)	787	236.0
Male	355 216 (51.6)	850	239.3
Age, y			
Mean (SD)	8.3 (5.8)	NA	NA
Newborn (<29 d)	47 372 (6.9)	69	145.7
29-364 d	33 210 (4.8)	368	1108.1
1-5	179 614 (26.1)	457	254.4
6-11	198 150 (28.8)	242	122.1
12-15	131 581 (19.1)	233	177.1
16-18	98 778 (14.3)	268	271.3
Days under observation, mean (SD)^c^	521.8 (107.3)	NA	NA
Household employment status			
Employed	402 196 (58.4)	856	212.8
Low-wage employment	40 867 (5.9)	105	256.9
Unemployed			
Short-term	15 004 (2.2)	40	266.6
Long-term	198 185 (28.8)	573	289.1
Other, not employed	32 453 (4.7)	63	194.1
Area-level median income			
Very low	5120 (0.7)	15	292.9
Low	123 795 (17.9)	495	399.9
High	124 329 (18.1)	240	193.0
Very high	435 461 (63.2)	887	203.7
Area-level mean living space per inhabitant			
Low	409 974 (59.5)	1067	260.3
Medium-low	134 804 (19.6)	294	218.1
Medium-high	134 319 (19.5)	257	191.3
High	9608 (14.0)	19	197.8
Urbanization			
City	413522 (60.0)	1062	256.8
Towns and suburbs	234 870 (34.1)	497	211.6
Rural area	40 313 (5.8)	78	193.5
Nationality			
German	522 755 (75.9)	1282	245.2
Non-German	165 950(24.1)	355	213.9
Comorbidities			
Obesity and other hyperalimentation	38 859 (5.6)	101	259.9
Diabetes	1748 (0.3)	18	1029.7
Congenital malformations of the circulatory system	13 028 (1.9)	101	775.3
Neoplasms	26 093 (3.8)	90	344.9
Asthma	46 145 (6.7)	148	320.7
Use of immunosuppressants	736 (0.1)	17	2309.8
Preexisting diseases, No.			
0	575 275 (83.5)	1229	213.6
1	100 993 (14.7)	349	345.6
≥2	12 437 (1.8)	59	474.4

Abbreviation: NA, not applicable.

^a^ Cases during the observation period from January 1, 2020, to July 13, 2021.

^b^ Rate was unstandardized.

^c^ During the observation period.

The incidence of COVID-19 hospitalization varied with age, with a low incidence among newborns, a high incidence among infants, and another peak among participants aged 16 to 18 years. However, the age-specific incidence should be interpreted with caution because of the different assignment dates of age in case and control participants. Regarding our main exposure, most children and adolescents came from households with a gainfully employed insurance holder; however, the proportion of long-term unemployed was particular high (198 185 [28.8%]). This distribution may be associated with a particular enrollment strategy in German statutory health insurance.

The crude incidence rates were lowest among participants from households with an insurance owner in gainful employment. Incidence gradually increased from low-wage employment to short- and long-term employment, with an incidence rate among long-term unemployed that was 51.4 cases per 100 000 higher than the overall incidence. Participants with lower area-level income, smaller mean living space, and higher degree of urbanization had a higher incidence of COVID-19 hospitalization. A slightly higher incidence was also recorded for German compared with non-German citizens. As expected, all comorbidities had incidence rates greater than the overall rate.

Descriptive results were reviewed with adjusted logistic regression models (Table 2). The trends for an association between the main exposure and a COVID-19 hospital diagnosis were stable after adjustment for sex, age, and time under observation. The same was observed for an adjustment of additional socioeconomic variables (model 3). Children of parents with long-term unemployment had the highest odds of COVID-19 hospitalization of all parental employment categories (OR, 1.36; 95% CI, 1.22-1.51), and children whose parents had low-wage employment also had an increased risk (OR, 1.29; 95% CI, 1.05-1.58). Importantly, additional adjustment for comorbidities (model 4) had almost no influence on the main estimates. The reason was that most diagnoses showed only weak or no association with household deprivation (eTable 1 in the Supplement).

**Table 2.** Associations Between Employment Status and COVID-19 Hospitalization, With 1637 Cases Among 688 705 Children and Adolescents Enrolled With a Mandatory Health Insurance Carrier in Germany

Factor	OR (95% CI)			
	Model 1^a^	Model 2^b^	Model 3^c^	Model 4^d^
Household employment status				
Employed	1 [Reference]	1 [Reference]	1 [Reference]	1 [Reference]
Low-wage employment	1.21 (0.99-1.48)	1.29 (1.05-1.58)	1.29 (1.05-1.58)	1.28 (1.04-1.57)
Unemployed				
Short-term	1.25 (0.91-1.72)	1.24 (0.90-1.70)	1.23 (0.89-1.69)	1.21 (0.88-1.67)
Long-term	1.36 (1.22-1.51)	1.38 (1.24-1.54)	1.36 (1.22-1.51)	1.35 (1.21-1.50)
Other, not employed	0.91 (0.71-1.18)	0.98 (0.76-1.27)	0.95 (0.74-1.23)	0.94 (0.73-1.22)
Gender				
Female	NA	1 [Reference]	1 [Reference]	1 [Reference]
Male	NA	1.02 (0.92-1.12)	1.02 (0.92-1.12)	1.01 (0.92-1.12)
Age (continuous)	NA	0.73 (0.71-0.76)	0.73 (0.71-0.76)	0.73 (0.71-0.75)
Age (quadratic)	NA	1.02 (1.02-1.02)	1.02 (1.01-1.02)	1.02 (1.02-1.02)
Mean days under observation	NA	1.00 (0.99-1.00)	1.00 (0.99-1.00)	0.99 (0.99-1.00)
Area-level household income				
High	NA	NA	1 [Reference]	1 [Reference]
Medium-high	NA	NA	1.40 (1.20-1.64)	1.42 (1.22-1.66)
Medium-low	NA	NA	3.22 (2.82-3.67)	3.30 (2.89-3.76)
Low	NA	NA	3.02 (1.73-5.28)	3.10 (1.78-5.43)
Area-level living space per inhabitant				
High	NA	NA	1 [Reference]	1 [Reference]
Medium-high	NA	NA	1.38 (0.84-2.28)	1.40 (0.84-2.31)
Medium-low	NA	NA	1.75 (1.06-2.89)	1.77 (1.07-2.92)
Low	NA	NA	3.07 (1.81-5.22)	3.12 (1.83-5.29)
Urbanization				
Rural area	NA	NA	1 [Reference]	1 [Reference]
Towns and suburbs	NA	NA	1.21 (0.94-1.55)	1.20 (0.94-1.54)
Cities	NA	NA	1.22 (0.90-1.65)	1.22 (0.90-1.65)
Nationality				
German	NA	NA	1 [Reference]	1 [Reference]
Non-German	NA	NA	0.95 (0.84-1.08)	0.99 (0.88-1.12)
Comorbidities				
Obesity and other hyperalimentation	NA	NA	NA	1.34 (1.09-1.65)
Diabetes	NA	NA	NA	4.48 (2.78-7.22)
Congenital malformations of the circulatory system	NA	NA	NA	3.11 (2.53-3.82)
Neoplasms	NA	NA	NA	1.44 (1.16-1.79)
Asthma	NA	NA	NA	1.69 (1.42-2.01)
Intake of immunosuppressants	NA	NA	NA	10.09 (6.17-16.52)

Abbreviations: NA, not applicable; OR, odds ratio.

^a^ Crude model.

^b^ Adjusted for sex, age, and time under observation.

^c^ Adjusted for sex, age, time under observation, and sociodemographic covariates.

^d^ Adjusted for sex, age, time under observation, sociodemographic covariates, and comorbidities.

The covariates sex, days under observation, degree of urbanization, and nationality were not independently associated with the outcome in the fully adjusted model, whereas all other variables were. Children living in areas with the lowest median income had 3-fold higher odds of COVID-19 hospitalization compared with children living in areas with the highest income (OR, 3.02; 95% CI, 1.73-5.28). Results were similar for living space. All comorbidities were associated with the outcome, but with different strengths. Use of immunosuppressants and diabetes were associated with highest risks of COVID-19 hospitalization (immunosuppressants: OR, 10.09; 95% CI, 6.17-16.52; diabetes: OR, 4.48; 95% CI, 2.78-7.22).

We conducted 2 sensitivity analyses. First, we repeated the analyses shown in Table 2 for a U07.1 diagnosis (laboratory-confirmed COVID-19 hospital diagnosis) only (Table 3). Crude estimates were comparable with the previous analyses with the exception of higher odds for children of low-wage employed insurance holders (OR 1.45; 95% CI, 1.08-1.96). In contrast to the main analyses, we observed that an adjustment for additional socioeconomic characteristics resulted in a slight reduction of odds for the main exposure variable.

**Table 3.** Associations Between Employment Status and 655 Laboratory-Confirmed COVID-19 Hospitalizations Among 688 705 Children and Adolescents Enrolled With a Mandatory Health Insurance Carrier in Germany

Household employment status	OR (95% CI)			
	Model 1^a^	Model 2^b^	Model 3^c^	Model 4^d^
Employed	1 [Reference]	1 [Reference]	1 [Reference]	1 [Reference]
Low-wage employment	1.45 (1.08-1.96)	1.69 (1.26-2.28)	1.52 (1.13-2.06)	1.52 (1.23-2.06)
Unemployed				
Short-term	1.19 (0.71-1.99)	1.20 (0.72-2.02)	1.18 (0.70-1.97)	1.16 (0.69-1.95)
Long-term	1.34 (1.13-1.58)	1.41 (1.19-1.67)	1.27 (1.06-1.51)	1.26 (1.06-1.50)
Other, not employed	1.02 (0.70-1.51)	1.08 (0.74-1.60)	1.04 (0.71-1.54)	1.03 (0.70-1.52)

Abbreviation: OR, odds ratio.

^a^ Crude model.

^b^ Adjusted for sex, age, and time under observation.

^c^ Adjusted for sex, age, time under observation, and sociodemographic covariates (ie, nationality, urbanization, living space per inhabitant, and area-level mean income).

^d^ Adjusted for sex, age, time under observation, sociodemographic covariates, and comorbidities (obesity and other hyperalimentation, diabetes, congenital malformations of the circulatory system, neoplasms, asthma, and intake of immunosuppressants).

A further exploration was made by calculating stratified models (age, sex, number of comorbidities) for an association between the main exposure and the outcome (Figure; eTable 2 in the Supplement). Associations were similar for male and female participants. In the case of age, odds were elevated for long-term unemployed across all age groups; however, estimates varied moderately for the other exposure categories. Variations in relation to the number of comorbidities were also small and unsystematic. Formal tests of interaction were not significant for all 3 variables.

**Figure.**
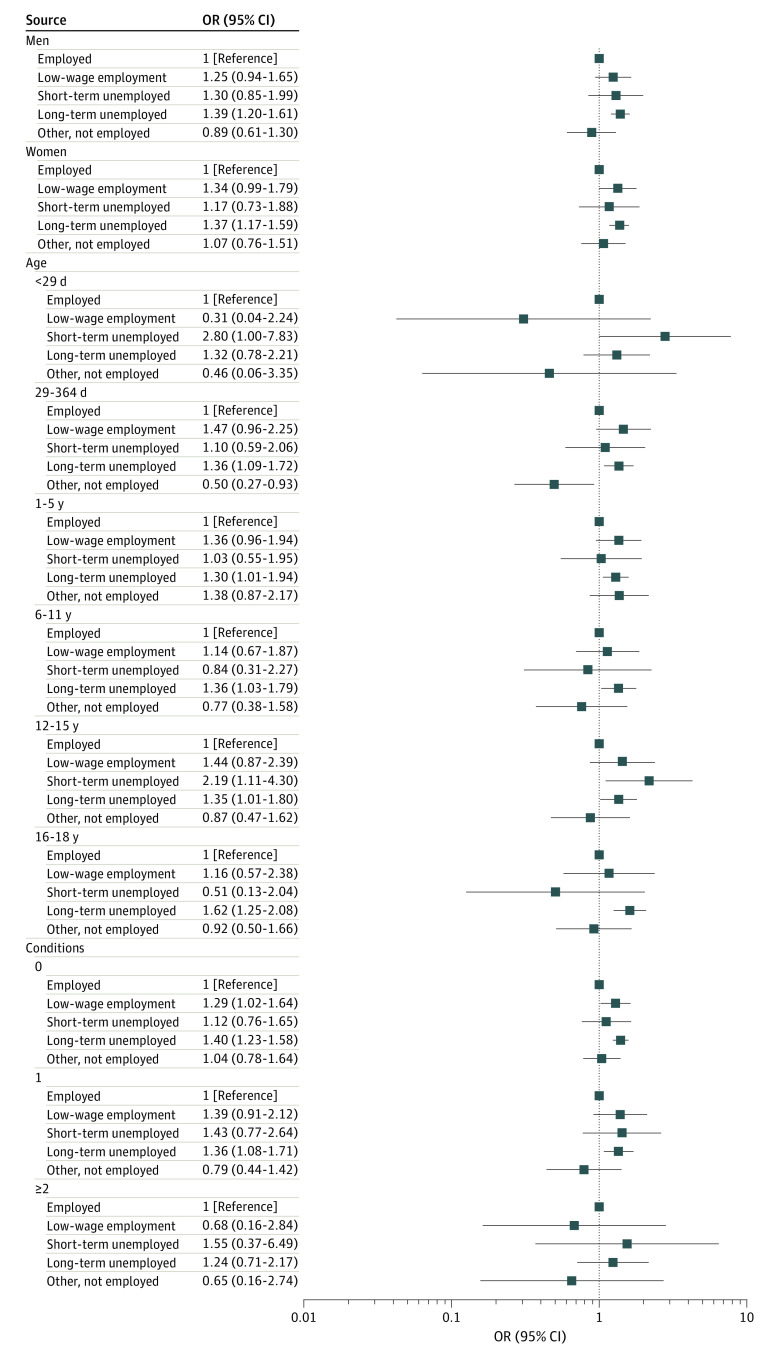
Association of Employment Status With COVID-19 Hospitalization Among 688 705 Children and Adolescents by Sex, Age, and Number of Comorbidities COVID-19 hospitalization was defined as *International Statistical Classification of Diseases and Related Health Problems, Tenth Revision, *Germany codes U07.1 and U07.2. OR indicates odds ratio.

## Discussion

Children and adolescents from households with an indication of poverty had elevated odds of a COVID-19 hospital diagnosis in this analysis of all insured people aged 0 to 18 years in a large German health insurance carrier. In addition to higher ORs for individual-level SEP, COVID-19 hospitalizations were also more frequent among those who lived in areas with lower median income and in districts with a smaller mean living space per inhabitant. These results support the assumption that social deprivation is a risk factor for a severe course of COVID-19. However, our hypothesis that inequalities in predisposing comorbidities are a main mediating mechanism was not confirmed.

Results on the association between SEP and COVID-19 hospitalizations are consistent with findings from studies with adults that showed higher COVID-19 hospitalization rates among disadvantaged populations.^8,14,15,16^ Only a few comparable investigations on pediatric hospitalization are available so far, and all used area-level proxy measures of individual SEP.^24,25,26,27,28^ The studies with the highest statistical power were 2 from England,^24,27^ which found higher hospital admission rates and higher pediatric intensive care unit admission rates among children from the most deprived regions. This is in line with our findings on area-level inequalities. Since COVID-19 shows a strong spatial clustering in terms of both incidence and severe courses,^6,7,8,37^ the concordant results suggest that some of the higher risks are associated with spatial characteristics, such as the characteristics of the inhabitants, local characteristics of the health care system, or infectious environments in low-income neighborhoods (eg, poor housing conditions, such as crowded conditions or a lack of infrastructure).^6,38,39,40,41^

However, no comparable study could be identified for our main exposure, ie, individual-level deprivation, and proxy measures of SEP predominate even among studies with adults. The categorization of household-level SEP used in this study allows for a more precise characterization of the individual socioeconomic situation, and the results suggest that deprivation is an individual risk factor for severe COVID-19 among children. This association was independent of age, sex, nationality, and characteristics of the area of residence. However, these findings are not unprecedented in previous research. A population-wide study of children and adolescents from Denmark, for example, which was conducted before the pandemic, showed that children from socioeconomically deprived families were more likely to be hospitalized than those from nondeprived families.^42^ This was true for several diagnoses, including respiratory and infectious diseases.

Our study, however, could not provide conclusive empirical evidence for the exact reasons for the association between SEP and COVID-19 hospitalizations. Based on previous studies, we assumed that children and adolescents from households with greater deprivation more often had preexisting diseases and, therefore, had an increased risk of a severe course.^33,34^ Comorbidities were indeed strongly associated with hospitalization. For example, use of immunosuppressants was associated with an approximately 10-fold higher risk, and diabetes was associated with an approximately 4.5-fold higher risk; these were the strongest risk factors in the model in general. These findings complement previous studies that found comparable patterns.^27,31,43,44,45^ However, an association with social inequality could not be established, as the diagnoses were only loosely associated with SEP. We think that certain childhood diseases may not be as sensitive toward social determinants as diseases later in life. The main mediating factors, such as social inequalities in major lifestyle risk factors (eg, smoking), are not yet present in early years. In addition, there is a higher etiological fraction of genetic predispositions in some of the diagnoses examined here (eg, congenital malformations of the circulatory system, childhood cancer). Studies on cancer, for example, found less evidence of an association of childhood cancer with inequalities compared with adult cancer.^46^

An alternative explanation for the individual inequalities found here could be the higher overall incidence rates of COVID-19 infections in children with vs without disadvantage, which could then result in higher hospitalization rates.^22,23,24^ Inequalities in infection risks among children could, on the one hand, reflect the known higher infection risks of adults in low-income households. On the other hand, specific exposures and susceptibilities may also be associated with child deprivation. It is known, for instance, that socioeconomic deprivation during early childhood and associated risk factors, such as malnutrition or stress, have a long-lasting negative effect on the immune systems of infants and children.^47,48^

Other risk factors leading to severe courses that were not included in this study are another possible explanation. Examples of these are passive smoking, physical inactivity, or poor diet.^38^ Lastly, differential use of the health care system may have played a role. Previous studies have shown that access to test facilities is not as good for deprived populations, which could have resulted in later diagnosis and worse status at the time of diagnosis.^9,49,50^ Moreover, hospital admission in Germany is often initiated by resident pediatricians and practitioners. Although studies are missing, it is conceivable that practitioners tend to admit children from vulnerable households more frequently to a hospital to ensure optimal health care.

Importantly, direct investigations into specific mediating pathways connecting COVID-19 infections and severity with socioeconomic deprivation in children and adolescents are very rare. Thus, many of the associations proposed in this study are speculative.

### Limitations and Strengths

This study has limitations. A major limitation is that information about outpatient diagnoses of COVID-19 was missing. This made it impossible to estimate the risk of detection bias, as we could not verify that the reason for hospitalization was a non–COVID-19 disease. Missing outpatient diagnoses also prevented the study of hospitalization rates among those with SARS-CoV-2 infection. An analysis of this rate would have added useful information about the processes leading from deprivation to infection to severe courses. Another limitation is that the measurement of chronic diseases relied on hospital diagnoses only in 2021. While it can be assumed that some diseases, such as neoplasms, usually lead to hospital stays, this may not be the case for obesity or asthma. Therefore, it is likely that the prevalence of chronic diseases was underestimated. Moreover, although it would have been possible to include additional diagnoses, we had to refrain from doing so due to very low prevalence rates (eg, of cystic fibrosis). Moreover, the district level was used to assign geospatial data. Although observations came from all 400 districts, the number of observations per unit varied because the insurance carrier mainly insures people living in western Germany. This could have reduced the validity of the area-level measures. It is noteworthy that most participants lived in highly urbanized regions, with a smaller living space and higher regional income than the German average. A further limitation is that we used data from a single insurance carrier. This particular carrier overrepresents people with unemployment, and our sample was, therefore, not representative of all young people in Germany with insurance.

The study also has some methodological strengths. The sample consisted of all eligible people enrolled with the insurance carrier. Thus, we avoided the risk of selection bias, which is common in data collected such that people with low SEP tend to have a significantly lower response rate. The high number of observations is another strength that made it possible to statistically evaluate such a rare outcome as COVID-19 hospitalizations among children. Additionally, all data came from official status reports with a very high validity. Lastly, the availability of an individual-level measure of SEP is another strength of this study.

## Conclusions

In this study, we found an association between the socioeconomic situation of children and adolescents and the risk of a COVID-19 hospital diagnosis. This suggests that greater attention must be paid to a possible severe course of the disease in children from vulnerable families and closer monitoring should be considered. In addition, the findings underline the need for socially sensitive prevention approaches at the societal level. However, we also found that the state of research on social inequalities in the pandemic, especially among children, has to be expanded. It is not clear, for example, which factors lead to children from disadvantaged families having more frequent COVID-19 hospital diagnoses. Even though severe clinical cases are less frequent in children than in adults, it is important to advance research in this field, given the vulnerability of the youngest.

## Notes

Supplement. **eTable 1.** Bivariate Associations Between Employment Status and Comorbidities Among 688 705 Children and Adolescents Enrolled With a Mandatory Health Insurance Carrier in Germany **eTable 2.** Logistic Regressions on Associations Between Employment Status and COVID-19 Hospitalization by Sex, Age, and Number of Preexisting Diseases Among 688 705 Children and Adolescents Enrolled With a Mandatory Health Insurance Carrier in Germany
