# Supplementary material for: Association of Household Deprivation, Comorbidities, and COVID-19 Hospitalization in Children in Germany, January 2020 to July 2021
Source: JAMA Netw Open. 2022 Oct 3;5(10):e2234319. doi: 10.1001/jamanetworkopen.2022.34319 (PMC9530965; doi:10.1001/jamanetworkopen.2022.34319)
Supplement: Supplement. — eTable 1. Bivariate Associations Between Employment Status and Comorbidities Among 688 705 Children and Adolescents Enrolled With a Mandatory Health Insurance Carrier in Germany eTable 2. Logistic Regressions on Associations Between Employment Status and COVID-19 Hospitalization by Sex, Age, and Number of Preexisting Diseases Among 688 705 Children and Adolescents Enrolled With a Mandatory Health Insurance Carrier in Germany [file jamanetwopen-e2234319-s001.pdf]

Supplemental Online Content

Dragano N, Dortmann O, Timm J, et al. Association of household deprivation, comorbidities, and COVID-19 hospitalization in children in Germany, January 2020 to July 2021. *JAMA Netw Open.* 2022;5(10):e2234319. doi:10.1001/jamanetworkopen.2022.34319

eTable 1. Bivariate Associations Between Employment Status and Comorbidities Among 688 705 Children and Adolescents Enrolled With a Mandatory Health Insurance Carrier in Germany

eTable 2. Logistic Regressions on Associations Between Employment Status and COVID-19 Hospitalization by Sex, Age, and Number of Preexisting Diseases Among 688 705 Children and Adolescents Enrolled With a Mandatory Health Insurance Carrier in Germany

This supplemental material has been provided by the authors to give readers additional information about their work.

© 2022 Dragano N et al. *JAMA Network Open.*

eTable 1. Bivariate Associations Between Employment Status and Comorbidities Among 688 705 Children and Adolescents Enrolled With a Mandatory Health Insurance Carrier in Germany

Numbers (percent) and p-values (based on Chi-squared tests)

	Employed	Low-wage employment	Short-term unemployed	Long-term unemployed	Other, not employed	p-values
Obesity and other hyperalimentation						0·001
No	381019 (94·7)	38206 (93·5)	14128 (94·2)	186242 (94·0)	30251 (93·2)	
Yes	21177 (5·3)	2661 (6·5)	876 (5·8)	11943 (6·0)	2202 (6·8)	
Diabetes mellitus						0·001
No	401183 (99·8)	40781 (99·8)	14969 (99·8)	197692 (99·8)	32332 (99·6)	
Yes	1013 (0·2)	86 (0·2)	35 (0·2)	493 (0·2)	121 (0·4)	
Congenital malformations of the circular system						0·40
No	394636 (98·1)	40059 (98·0)	14700 (98·0)	194430 (98·1)	31852 (98·2)	
Yes	7560 (1·9)	808 (2·0)	304 (2·0)	3755 (1·9)	601 (1·8)	
Neoplasms						0·001
No	385126 (95·7)	39604 (96·9)	14410 (96·0)	192255 (97·0)	31217 (96·2)	
Yes	17070 (4·2)	1263 (3·1)	594 (4·0)	5930 (3·0)	1236 (3·8)	
Asthma						0·001
No	374387 (93·1)	38169 (93·4)	13983 (93·2)	185846 (93·8)	30175 (93·0)	
Yes	27809 (6·9)	2698 (6·6)	1021 (6·8)	12339 (6·2)	2278 (7·0)	
Intake of immunosuppressants						0·12
No	401787 (99·9)	40833 (99·9)	14988 (99·9)	197950 (99·9)	32411 (99·9)	
Yes	409 (0·1)	34 (0·1)	16 (0·1)	235 (0·1)	42 (0·1)	
Number of comorbidities						0·001
0	334896 (83 3)	34054 (83·3)	12467 (83·9)	167159 (84·4)	26699 (82·2)	
1	60004 (14·9)	6111 (14·9)	2250 (15·0)	27553 (13·9)	5075 (15·6)	
2+	7296 (1·8)	702 (1·7)	287 (1·9)	3473 (1·8)	679 (2·1)	

© 2022 Dragano N et al. *JAMA Network Open.*

eTable 2. Logistic Regressions on Associations Between Employment Status and COVID-19 Hospitalization by Sex, Age, and Number of Preexisting Diseases Among 688 705 Children and Adolescents Enrolled With a Mandatory Health Insurance Carrier in Germany

	Low-wage employment<br><br>OR 95%CI Reference=employed	Short-term unemployed<br><br>OR 95%CI Reference=employed	Long-term unemployed<br><br>OR 95%CI Reference=employed	Other, not employed<br><br>OR 95%CI Reference=employed
Sex[b]				
Male	1·25 (0·94–1·65)	1·30 (0·85–1·99)	1·39 (1·20–1·61)	0·89 (0·61–1·30)
Female	1·34 (0·99–1·79)	1·17 (0·73–1·88)	1·37 (1·17–1·59)	1·07 (0·76–1·51)
Age groups[c]				
Newborn (<29 days)	0·31 (0·04–2·24)	2·80 (1·00–7·83)	1·32 (0·78–2·21)	0·46 (0·06–3·35)
29–364 days	1·47 (0·96–2·25)	1·10 (0·59–2·06)	1·36 (1·09–1·72)	0·50 (0·27–0·93)
1–5 years	1·36 (0·96–1·94)	1·03 (0·55–1·95)	1·30 (1·01–1·94)	1·38 (0·87–2·17)
6–11 years	1·14 (0·67–1·87)	0·84 (0·31–2·27)	1·36 (1·03–1·79)	0·77 (0·38–1·58)
12–15 years	1·44 (0·87–2·39)	2·19 (1·11–4·30)	1·35 (1·01–1·80)	0·87 (0·47–1·62)
16–18 years	1·16 (0·57–2·38)	0·51 (0·13–2·04)	1·62 (1·25–2·08)	0·92 (0·50–1·66)
Number of comorbidities[d]				
0	1·29 (1·02–1·64)	1·12 (0·76–1·65)	1·40 (1·23–1·58)	1·04 (0·78–1·64)
1	1·39 (0·91–2·12)	1·43 (0·77–2·64)	1·36 (1·08–1·71)	0·79 (0·44–1·42)
2 and more	0·68 (0·16–2·84)	1·55 (0·37–6·49)	1·24 (0·71–2·17)	0·65 (0·156–2·74)

[a] OR= odds ratio, 95%CI= 95% confidence interval
[b] estimates are adjusted for age in years, age², and days under observation
[c] estimates are adjusted for sex and days under observation
[d] estimates are adjusted for sex, age in years, age², and days under observation

© 2022 Dragano N et al. *JAMA Network Open.*
